# Allosterism of Nicotinic Acetylcholine Receptors: Therapeutic Potential for Neuroinflammation Underlying Brain Trauma and Degenerative Disorders

**DOI:** 10.3390/ijms21144918

**Published:** 2020-07-12

**Authors:** Swarup Mitra, Shailesh N. Khatri, Malabika Maulik, Abel Bult-Ito, Marvin Schulte

**Affiliations:** 1 Department of Pharmacology and Toxicology, The State University of New York at Buffalo, Buffalo, NY 14203, USA; 2 Department of Cellular and Integrative Physiology, University of Texas Health Science Center at San Antonio, San Antonio, TX 78229, USA; khatrisn@uthscsa.edu; 3 Department of Biochemistry and Neurology, Hunter James Kelly Research Institute, The State University of New York at Buffalo, Buffalo, NY 14203, USA; malabika@buffalo.edu; 4 Department of Biology & Wildlife, University of Alaska Fairbanks, Fairbanks, AK 99775, USA; abultito@alaska.edu; 5 Department of Biomedical and Pharmaceutical Sciences, Idaho State University College of Pharmacy, Pocatello, ID 83209, USA; schumarv@isu.edu

**Keywords:** neuroinflammation, nicotinic acetylcholine receptors, allosteric modulators

## Abstract

Inflammation is a key physiological phenomenon that can be pervasive when dysregulated. Persistent chronic inflammation precedes several pathophysiological conditions forming one of the critical cellular homeostatic checkpoints. With a steady global surge in inflammatory diseases, it is imperative to delineate underlying mechanisms and design suitable drug molecules targeting the cellular partners that mediate and regulate inflammation. Nicotinic acetylcholine receptors have a confirmed role in influencing inflammatory pathways and have been a subject of scientific scrutiny underlying drug development in recent years. Drugs designed to target allosteric sites on the nicotinic acetylcholine receptors present a unique opportunity to unravel the role of the cholinergic system in regulating and restoring inflammatory homeostasis. Such a therapeutic approach holds promise in treating several inflammatory conditions and diseases with inflammation as an underlying pathology. Here, we briefly describe the potential of cholinergic allosterism and some allosteric modulators as a promising therapeutic option for the treatment of neuroinflammation.

## 1. Introduction

Inflammation is the body’s defense mechanism that is pivotal for maintaining health status. Inflammation encompasses immune responses to noxious and harmful stimuli [1,2,3,4,5]. At the tissue level, inflammation involves the recruitment of plasma proteins, fluid and leukocytes into the damaged area resulting in vasodilation, a phenomenon characterized by a surge in blood flow, edema and increased vascular permeability [6]. Any damage signals are identified through transmembrane receptors such as Toll-like receptors (TLRs), the intracellular nucleotide-binding domain and leucine-rich-repeat-containing receptors (NLRs) [7,8,9]. Activation of these receptors often culminates in the potentiation of a critical transcription factor, the nuclear factor kappa-light-chain-enhancer of activated B cells (NF-κB) that leads to translocation of NF-κB into the nucleus for promoting the transcription of target genes. Transcription and translation enable the expression of a set of these target genes that are mostly pro-inflammatory cytokines such as interleukins (ILs), tumor necrosis factor-alpha (TNF-α) and others [10]. Inflammatory cytokines further coordinate with various cellular intermediates facilitating the recruitment of effector cells such as neutrophils and monocytes in the periphery and microglia and astrocytes in the central nervous system (CNS) [11]. These effector cells in turn undergo degranulation releasing reactive oxygen species (ROS), reactive nitric oxide species (RNS) and proteinases that promote the inflammatory process [11]. While infections are the primary factors underlying inflammation, injury or trauma and exposure to foreign matter can trigger inflammatory responses [12]. Emerging evidence shows that aging and environmental factors such as lifestyle, food habits and exercise can influence inflammation [13,14,15,16,17].

Inflammation is the underlying cause of several pathological conditions that impact more than 500 million patients across major pharmaceutical markets [2]. Despite significant strides in the development of therapeutic strategies against various inflammatory diseases, the major public health concern has been the link to a more persistent issue of chronic inflammation [2]. There is accruing evidence of the role of neuronal cholinergic mechanisms influencing neuroinflammatory cascades implicated in trauma and debilitating nervous system manifestations, such as clusters of neurodegenerative disorders, autoimmunity, and chronic nociception [4,18,19,20,21].

Historically, the cholinergic anti-inflammatory pathway emerged with the discovery of T-lymphocyte cytotoxicity via the muscarinic cholinergic system [22]. These claims were further corroborated in experiments demonstrating inhibition of T-cell differentiation and responses due to nicotine exposure [23,24]. Recent evidence points towards a nAChR mediated cholinergic regulation of inflammation in the CNS [2]. For example, the cholinergic anti-inflammatory pathway involving activation of nAChRs on the vagus nerve can inhibit cytokine release, thereby preventing their damaging effects, and has become an essential link between CNS and immunomodulation in response to immune challenges [25,26]. Recent studies have also shown that the cholinergic anti-inflammatory mechanisms extend beyond the aforementioned pathway involving discrete cascades in the CNS. α7 nAChR expression on primary dorsal root ganglion (DRG) neurons influences excitatory glutamatergic-signaling-mediating spinal analgesia [27,28,29,30]. Similarly, activation of α7 nAChR on astrocytes attenuates LPS-mediated upregulation of inflammatory cytokines through inhibition of the ubiquitous NF-κB inflammatory pathway [31]. Additionally, α7 involves phosphorylation of STAT3, a mediator of an anti-apoptotic cascade under inflammatory challenge conditions [32].

Pharmacological targeting of nAChRs has therefore gained traction in recent years to address its role in inflammation. Despite successful drug design, the molecules designed to target primary active (orthosteric) sites often have limitations, as these sites are highly conserved [33]. Thus, drugs designed for this site often end up having overlapping specificity. On the contrary, drugs targeted to the secondary (allosteric) binding sites carry greater pharmacological potential [33]. This review emphasizes the potential of cholinergic allosterism as an emerging approach to drug design for the treatment of chronic neuroinflammation. We predominantly focus on traumatic brain injury (TBI) and major neurodegenerative disorders, Alzheimer’s (AD), Parkinson’s (PD) and Multiple sclerosis (MS) in which inflammation forms a significant pathological basis [34]. We highlight the role of nAChRs in influencing the neuroinflammatory mechanisms underlying these pathophysiologies and how allosteric targeting of some of the nAChRs subtypes can formulate a potential therapeutic strategy for successfully treating these disorders.

## 2. Nicotinic Acetylcholine Receptors and Allosterism

The nAChRs are members of the Cys-loop superfamily of receptors that include GABAA, glycine and serotonin receptors [35,36,37]. nAChRs are large pentameric membrane-bound proteins of a molecular mass of ~290 KD. Each receptor is formed by combinations of five identical (i.e., homomeric) or different (i.e., heteromeric) subunits. The subunits that contribute to nAChRs are α (α2–α9) and β (β2–β4) [35]. The diversity of subunit configuration and assembly results in a myriad of receptor subtypes with varied sensitivity to ligands. Adding to the complexity and variability of nAChRs, each heteromeric receptor may potentially exhibit different stoichiometry based on the ratio of α:β subunits present and varied sensitivities to endogenous ligand (acetylcholine, ACh) [38]. One of the widely studied nAChRs and their stoichiometries are the α4β2 receptors, which comprise of high sensitive (HS)(α4)2(β2)3 and low sensitive (LS)(α4)3(β2)2 receptor organization [39,40]. Such stoichiometric variation coupled with context-dependent intrinsic biophysical properties and participation of other α or β subunits imparts unique heterogeneity in the physiological functionality of the nAChRs [38]. The predominant nAChR subtypes expressed in the CNS comprise of α4 and β2 subunits [36,41]. Additionally, α6β2 and α7 subtypes are abundantly found in the CNS, while α6β4 exhibit limited distribution [42]. Post-synaptically, they facilitate fast cholinergic neurotransmission, and pre-synaptically they modulate the release of other neurotransmitters and, therefore, play diverse roles ranging from cognition to modulation of neurotransmitters, and neuroprotection [43].

nAChRs are fast desensitizing ligand-gated ion channels that are activated by ligands binding at the orthosteric site [44,45,46]. Most therapeutic ligands are targeted towards the orthosteric site where they alter synaptic neurotransmission and receptor expression profiles. Thus, prolonged activation of nAChRs at the orthosteric site often results in tolerance or insensitivity to the drug [47], suggesting that suboptimal or non-orthosteric activation may be beneficial. Despite the layers of heterogeneity in nAChRs exhibited by its subunits, stoichiometries and biophysical properties, the ACh binding site, where all agonist, partial agonist or antagonist bind, is highly conserved [48]. This posits a limitation in the development of a clinically usable new ligand with adequate specificity across diseases in which nAChRs are involved, including inflammation. Hence, besides tolerance/insensitivity, ligands developed for orthosteric sites lack specificity and may lead to potential side effects [49]. However, allosteric ligands present a great potential, as they act through binding to a non-orthosteric site and activate nAChRs only in the presence of endogenous ligands such as ACh [49].

By definition, allosteric ligands bind to non-orthosteric sites and may possess intrinsic activity or may be ineffective by themselves, i.e., possess no intrinsic activity. The allosteric ligands that possess no intrinsic activity are also referred to as allosteric modulators (AMs) [33,49,50]. The number of allosteric sites present, and their structural diversity exclusively depends on the subunit type and the subunits that assemble structurally to form a functional receptor [51,52,53,54,55,56]. This allows diversity on the allosteric site, as well as potential tunability of nAChRs to produce greater specificity. Allosteric modulators/ligands can either enhance or reduce the nAChR responses induced by ACh [47]. Ligands that enhance the ACh responses are positive allosteric modulators (PAMs), whereas those that reduce ACh responses are known as negative allosteric modulators (NAMs) [47]. Allosteric modulators may impart their effects on ACh-induced responses by inducing a change in ACh potency or by altering efficacy and/or receptor opening probability without affecting baseline neurotransmission [47,57].

Different types of PAMs targeting α7 nAChRs are studied and classified as type I and II, based on the mechanism by which they modulate the receptors [58]. The type I PAM increases Ach-induced current amplitudes, potentially through increasing ACh potency and efficacy, with minimal effect on receptor kinetics. Type II PAMs, however, work by altering receptor kinetics, specifically by slowing the desensitization and deactivation, leading to prolonged activation of receptors [59]. Another class of PAMs exists that exhibit intrinsic activity, i.e., they can directly activate the receptors. PAMs that potentiate orthosteric agonist-induced currents and possess intrinsic activity are called agonist-PAMs or Ago-PAMs [60]. For detailed accounts of allosteric modulation, underlying mechanism and receptor states please refer to these reviews [61,62,63].

## 3. Neuroinflammation and Potential of Cholinergic Allosterism

In the brain, chronic inflammation is a persistent cellular anomaly that is associated with several neurological disorders [64]. Despite having divergence in inflammatory inducers specific to each disease, there is convergence in mechanisms underlying amplification of inflammatory processes [64]. Under optimal physiological conditions, microglia, the resident immune cells of the brain, exhibit a deactivated phenotype [65]. Alteration to a more activated inflammatory phenotype occurs in response to an immune stimulus [64]. A sustained stimulus disrupts the homeostatic balance in the inflammatory mechanisms inflicting a chain of events that often results in neurotoxic challenges involving ROS, RNS and pro-inflammatory cytokines manifesting into cytokine-mediated diseases [66,67]. Additionally, the neurotoxic events often cause reactive astrocytosis, increased vascular permeability, extravasation of proteins, blood–brain barrier alterations and axonal demyelination that aid in amplifying the underlying disease states [64]. Neuroinflammation forms an integral aspect of some of the most devastating neurological disorders that will be discussed in the following sections.

### 3.1. Alzheimer’s Disease (AD)

AD is an age-related devastating neurodegenerative condition that progressively impairs cognition, affective functionality and social well-being [68]. At the pathological level, AD is characterized by β-amyloid plaques and neurofibrillary tangles and symptomatically often involves dementia, speech impairments and disorientation, eventually leading to loss of self-care and death [68,69,70]. Epidemiological observations and postmortem analysis have corroborated inflammatory dysregulation as a major contributing factor to the disease state [71,72]. Decades of research have implicated various inflammatory mediators in the onset and progression of a persistent neuro-inflammatory cycle that initiates and exacerbates the AD pathology [73,74,75,76,77,78,79,80,81,82]. Age-related oxidative imbalance that might be further influenced through environmental triggers is often the starting point that leads to ROS and RNS production through the concerted action of several inflammatory cytokines such as IL-1, IL-6, TNF-α and oxidative enzymes such as NADH oxidase and nitric oxide synthase [69,73,74,75,76,77,78,79,80,81,82,83,84,85]. The disproportionate ratio of the oxidative species can further lead to exaggerated cytokine responses. Such incremental cytokine production over a period of time can synergistically produce distress signals to the astrocytes and microglia, which express more cytokines producing an inflammatory hotspot [86]. These anomalous mechanisms coupled with a genetic predisposition for AD disrupt the overall cellular homeostatic balance towards a more pathological phenotype [87].

Cholinergic hypofunction typical of AD is an established phenomenon and loss of cholinergic pathways is an important contributor to dementia pertaining to attention, spatial and episodic domains [88,89]. Using acetylcholinesterase (AChE) inhibitors has, therefore, proven to be one of the most viable therapeutic options for symptomatic improvement [90]. There is evidence on the role of nAChRs in mediating inflammatory mechanisms underlying AD [91]. The interactions of the cholinergic system and a ubiquitous neurotrophin, nerve growth factor (NGF) in the manifestation of AD is a well-known phenomenon [92,93,94,95,96]. The cholinergic neurons of the basal nucleus of Meynert are deprived of trophic support due to dysregulated retrograde transport of NGF. Such disruption of NGF transport mechanisms in conjunction with cholinergic transmission exacerbates β-amyloid mediated toxicity in cholinergic neurons [97,98,99,100]. Since NGF has an established role in influencing inflammatory mechanisms [101], the intertwining role of NGF and cholinergic transmission at the level of receptor pharmacology warrants investigation. Further, postmortem analysis has demonstrated up to a 50% reduction in α4β2 nAChRs in the brain of AD patients [102], early in pathogenesis [103]. Additionally, both α4 and α7 subunits exhibit reduced expression in autopsy samples of the human cerebral cortex of AD patients [104,105,106]. With such extensive involvement of the cholinergic system in AD pathophysiology, one of the pressing questions is regarding the pharmacological modulation of nAChRs in influencing the disease state. In-vitro administration of nicotinic agonists in PC12 cells and rat cortical neurons inhibits β-amyloid associated toxicity while chronic nicotine administration in a transgenic AD mouse model attenuates β-amyloidosis and neurite atrophy by decreasing astrogliosis, a phenomenon typical of neuroinflammation [107,108,109,110]. The neuroprotective mechanism against β-amyloid toxicity is mediated through the α7-Janus kinase 2 (JAK2) pathway that involves the association of JAK2 with α7 nAChR subsequently activating phosphoinositide 3-kinase (PI3K) and protein kinase B (Akt) phosphorylation [107,111,112]. These findings have further been supported through findings in the AD mouse model implicating an α7-regulated pro-survival cascade involving mitogen activated protein kinase (MAPK), Bcl2 and NF-κB. Blocking α7 receptors by specific antibodies causes neuroinflammation leading to Alzheimer’s-like symptoms in rodents [91]. Additionally, the interactions of α7 nAChRs with β-amyloid have been well documented [113,114,115] implicating nAChRs and their pharmacological targeting as a critical avenue for therapeutic value in AD.

### 3.2. Parkinson’s Disease (PD)

PD is a neurodegenerative disorder that progressively worsens with age [116]. Aggregation of α-synuclein in cells of the substantia nigra in the brain and loss of dopaminergic neurons are the major pathological hallmarks [117,118,119]. PD is associated with impaired movement and reduced cognitive function [120,121]. The etiology of PD can be attributed to a combination of environmental and genetic factors [122,123,124,125]. Nicotinic neuroprotection in PD is evidenced through several experimental studies [126,127,128,129]. Nicotine protects against nigrostriatal lesions caused by 6-hydroxydopamine (6-OHDA) and 1-methyl-4-phenyl-1,2,3,6-tetrahydropyridine (MPTP) insults that recapitulate PD-like pathology [127,130,131,132,133,134]. 6-OHDA and MPTP produce neuronal atrophy through oxidative stress and exacerbated microglial activation mechanisms, which are essential for neuroinflammation [135]. Therefore, the role of nAChRs becomes critical in PD. The striatal nAChR subtypes (including α4β2) are also reduced due to nigrostriatal damage [136,137]. Further, post-mortem brain analysis of PD patients has revealed neuroinflammation as a contributing factor in α-synuclein mediated neurotoxicity and PD patients display higher striatal levels of TGF-β, IL-1β, IL-6, IFN-γ and IL-1 [138,139,140,141,142]. Pharmacological targeting of both α4β2 and α7 receptors [132,133,143] has shown neuroprotective effects in 6-OHDA-lesioned rats by partially modulating Parkinson’s pathology, while α4 knockout does not show nicotine-mediated neuroprotection of dopaminergic neurons [144] emphasizing the importance of nAChRs in modulating pathological endpoints. Moreover, nicotine administered both before and after 6-OHDA insult was shown to be more effective against partial, but not complete dopaminergic lesions in the substantia nigra [130].

In addition to the conventional nAChR subtypes, there is emerging evidence of the importance of α6 and α5 subunits in regulating dopaminergic transmission [145,146]. Recent research implicates cellular calcium imbalance [147,148] as a probable mechanism underlying nAChR regulation. nAChR-mediated calcium alterations lead to signal transduction involving PI3K and or JAK2/STAT3 signaling that eventually alters inflammatory endpoints and pro-survival cascades [148].

### 3.3. Multiple Sclerosis (MS)

MS is an inflammatory autoimmune disorder that is characterized by chronic inflammatory demyelination and axonal degeneration in the CNS [149]. This process occurs both in white matter and grey matter [150], which results in disruption of axonal transmission causing motor and sensory impairments [149]. Though the precise molecular events that lead to MS are unknown, inflammatory anomalies, a key phenomenon in the disease pathology, result in the breakdown of the blood–brain barrier vascular endothelium affecting myelin and oligodendrocytes [151,152]. Further, activation of lymphocytes, macrophages, dendritic cells, and microglia are observed at the onset of the disease [153,154]. A cholinergic role in neuroinflammation and MS is evident by the presence of acetylcholinesterase (AChE), an enzyme regulating ACh turnover, and its variants in neurons, blood cells, white matter, glia and lymphocytes [155,156,157]. Nicotine has been shown to inhibit experimental MS in rodents [158,159], suggesting immune function could be manipulated by targeting cholinergic pathways of immune cells by specific ligands, potentially AMs [160,161]. The most promising target thus far has been α7 nAChR. The immune cells expressing α7 also express a protein called RIC-3 (resistance to inhibitors of cholinesterase 3) that aids in the surface expression of α7 nAChR, influencing the disease state and are being considered as cholinergic targets of MS [162]. Additionally, neuroinflammation induced by lipopolysaccharide (LPS) insult in rats was inhibited through activation of α7 nAChR [163]. Inhibition of AChE is shown to curb inflammation by reducing lymphocyte proliferation and secretion of proinflammatory cytokines such as TNF-α, IL-B and IL-6 in mice, indicating that targeting AChE may provide a positive outcome in the treatment of MS [149,164,165]. In the experimental autoimmune encephalomyelitis (EAE) mouse model of human MS, choline acetyltransferase (ChAT) expression, an indicator for ACh presence/synthesis, in natural Killer (NK) cells is observed. ChAT positive cells promote migration of NK cells into the CNS, ameliorating the disease severity [166,167]. It was found that mature NK cells expressing ChAT were able to repress EAE induction and had a greater capacity to delay disease onset and decrease symptom severity compared to NK cells devoid of ChAT [168,169]. Using an agonist of α7 nAChRs in EAE showed that the activation of α7 is necessary for the suppression of EAE clinical severity [170]. These findings support other accounts that higher serum levels of ACh are observed in MS patients receiving treatments [171,172]. Hence there is substantial evidence suggesting that increasing cholinergic signaling in MS patients may ameliorate their symptoms.

### 3.4. Traumatic Brain Injury (TBI)

TBI is due to injury from an external force and is a major human health concern that impacts people of all ages [173]. TBI poses a major socioeconomic burden with devastating long-term consequences [174]. External trauma results in leakage of the blood–brain barrier (BBB) that initiates the infiltration of inflammatory cells [175,176,177]. Preclinical studies have established that TBI dysregulates cholinergic mechanisms that are characterized by reductions in choline uptake, choline acetyltransferase activity and vesicular acetylcholine transporter activity [178,179,180,181]. At the receptor level, α7 receptor expression is attenuated by 50% [181]. These results have further been substantiated in human TBI studies [182,183,184]. More recent evidence is emerging on the role of nAChRs in modulating the inflammatory profile in TBI [185,186]. nAChR α7 null mice exhibit potentiated levels of TNF-α and IL-β levels concomitant with a leaky BBB [156]. Whereas systemic administration of the nAChR α7 agonist PNU-282987 or the positive allosteric modulator PNU-120596 significantly attenuates TBI-triggered BBB compromise [156]. Further, quantitative autoradiography in TBI rats shows reduced α7, α4 and α3 binding in various brain regions and activating α7 results in a dampened inflammatory cytokine response [187,188].

## 4. Potential Allosteric Modulators as Therapeutics

The development of AMs-based therapeutics targeting nAChRs remains challenging because of the heterogeneous and unique role of nAChRs in promoting neuroinflammation. However, several selective and non-selective AMs are available that can be repurposed as lead molecules to design promising candidates. We believe the greater strategy would be to employ deconstruction-reconstruction (D-R) approaches to the existing AMs, generate analogs and screen them for positive or negative allosteric modulation. Briefly, the D-R approach involves fragmentation of known ligands, where each fragment can serve as a key pharmacophore. Upon analysis and optimization, a suitable fragment that enables orthosteric ligand to stabilize active (open) receptor conformation is selected for reconstruction. Reconstruction is relatively challenging as it not only calls for merging, linking or growing fragments to develop drugs but also to adhere to classical guidelines for maintaining physicochemical properties such as LogP, topological polar surface area, molecular weight, etc. [189,190,191].

Desformylfulstrabromide (dFBr) is a PAM of neuronal α4β2 receptors, first extracted from the bryozoan Flustra foliacea [192]. dFBr exhibits a bell-shaped dose-response curve where it enhances ACh induced currents in α4β2 receptors at lower concentrations and inhibits them at higher concentrations [193]. At potentiating concentrations, dFBr is thought to rescue the receptor from the desensitizing state leading to greater potentiation [193]. dFBr does not show any potentiation of nAChRs containing α3 or α7 receptors [52,194]. In rats and mice, dFBr was shown to reduce intravenous nicotine self-administration without supporting self-administration behavior [195] and compulsive-like behavior [196,197], respectively. In mouse models of neuropathic pain, dFBr potentiates antiallodynic responses of nicotine [198], suggesting that dFBr can be used in combination with an agonist or partial agonist to enhance or maintain cholinergic tone. In the same study, dFBr failed to affect allodynia when solely injected on its own. In vivo and in vitro activation of α4β2 nAChRs on mouse inflammatory macrophages by agonists are shown to alleviate inflammation-mediated neuropathic pain [199]. Also, dFBr has been found to relieve β-amyloid peptide (Aβ1–42) mediated loss of α4β2 function in oocytes [200]. This further solidifies the role of α4β2 and the potential of dFBr as a lead therapeutic molecule.

NS9283 is a selective PAM of α4β2 receptors, developed at Neurosearch Inc. Specifically, it enhances ACh-induced currents of LS stoichiometry [55,56]. Analogs of NS9283 have been shown to be selective for other variants of the receptor, including α4α5β2 [201]. Given the potential involvement of α5-containing receptors in neuroinflammation, these analogs of NS9283 may be valuable leads in developing selective and therapeutically useful anti-inflammatory AMs.

NS206 is a PAM selective for the α4 subunit [56] and it potentiates both the LS and HS stoichiometry. NS206 has similar potencies for α4β2 and α4β4 receptors but does not potentiate α3β4 or α7 receptors. The ability to potentiate HS α4β2 receptors is of particular advantage as they are thought to be involved in neurological disorders including neuroinflammation [56,202]. Unlike NS9283, NS206 enhances ACh efficacy rather than potency [203]. Both NS206 and NS9283 are selective for the α4 subunit, however, their binding sites have been mapped to different domains of the same subunit. When co-applied, their effects are additive [203].

Galantamine is a plant-based alkaloid from the amaryllis family [204]. It was first identified as an anti-acetylcholinesterase and later found to be a type I PAM for nAChRs [205,206]. Galantamine has been used clinically for Alzheimer’s disease [207] and has also been explored as a potential therapeutic option for Autism [208]. Evidence suggests that galantamine is a PAM for human α4β2 and α7 receptors. Galantamine was shown to enhance ACh responses by 22% in α7 receptors at lower concentrations and reduce them at higher concentrations, producing a bell-shaped dose-response curve [209], a typical feature of most PAMs. As galantamine exhibits both PAM and NAM activity at different concentrations, it is possible to construct analogs possessing either PAM or NAM activity without acetylcholinesterase activity [210]. This notion is further substantiated by a recent finding that galantamine’s [211] and a selective agonist’s [212] anti-inflammatory effects are mediated by α7 receptors. Similarly, other acetylcholinesterase inhibitors such as physostigmine [213] can also be explored for cholinergic anti-inflammatory activity. A recent conflicting study suggested that galantamine is not a PAM of either α4β2 or α7 receptors expressed in Xenopus oocytes and HEK 293 cells [209]. Nevertheless, galantamine does provide a substrate for designing a specific PAM or NAM that could enable the optimal functioning of the nAChRs for recovering aberrant inflammatory activation. Furthermore, the PNU series of PAMs (e.g., PNU-120596) and Ago-PAM (GAT-107) for α7 receptors are shown to reduce nociceptive behavior, neuropathic pain and thermal hyperalgesia [214,215,216,217] and could be potential leads for developing AMs in treating neuroinflammation.

Levamisole is an effective anthelmintic that binds to nematode muscle nAChRs [218]. Due to side effects like severe dermatological lesions, its use in humans is curbed, however, they are still used in veterinary medicine [218,219,220]. In humans, levamisole has also been used as an adjuvant in colon cancer therapy [221] and is a common intentional contaminant of cocaine [222,223]. Levamisole was identified as a PAM of α3 containing nAChRs. It potentiates ACh-induced responses at α3β2 receptors at lower concentrations and inhibits them at higher concentrations [224]. It also acts as a partial agonist of α3β4 receptors at very high concentration. Levamisole poses several serious side effects including elicitation of inflammatory diseases [225], however, they are not explicitly attributed to its PAM activity on nAChRs. It is possible to design receptor subtype-specific analogs of levamisole while retaining its PAM activity and minimizing observed side effects. Further thorough research is warranted before levamisole can be developed as an anti-inflammatory therapeutic agent.

Another promising compound, HEPES, is a commonly used buffering agent, that is shown to selectively potentiate HS α4β2 while slightly inhibiting LS α4β2 receptors [39]. Likewise, several piperidines (e.g., CMPI) and their analogs have been identified as potent, as well as selective α4β2 nAChR PAMs [53,54].

## 5. Conclusions

**Figure 1:**
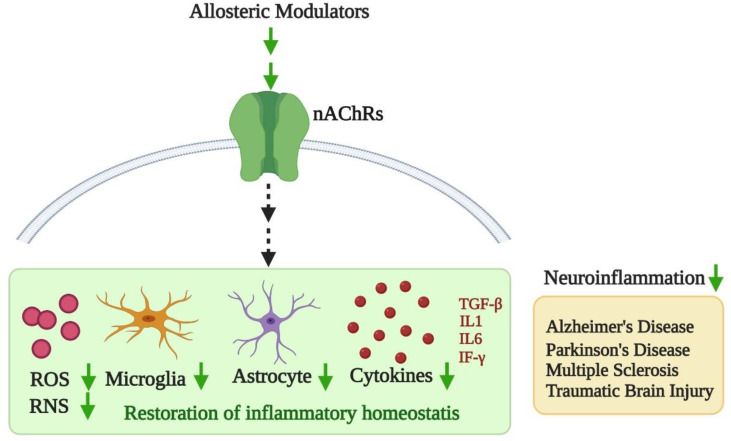
Proposed model of allosteric modulation in inflammation: Allosteric modulators can potentially restore cholinergic transmission in the cells by binding to neuronal acetylcholine receptors (nAChRs). This can attenuate the exacerbated expression of predominant inflammatory mediators such as reactive oxygen species (ROS), reactive nitric oxide species (RNS), astrocytes, microglia and cytokines such as TGF-β (Transforming growth factor-beta), interleukin 1 (IL-1), interleukin 6 (IL-6) and interferon-gamma (IF-γ).

Appropriate drug candidates and cellular targets to combat dysregulation of fundamental inflammatory pathways underlying numerous inflammatory driven neurodegenerative conditions are currently lacking. With chronic inflammation being implicated in several devastating diseases, such as AD, PD, MS and TBI, a thorough screening of some of the cellular mediators that lie at the crossroads of inflammation needs to occur. Mounting evidence shows that cholinergic mechanisms significantly overlap with inflammatory cascades and carry the potential to modulate the molecular mechanisms and functional outcomes of inflammation. Current research on the neuro- immunomodulatory role of nAChRs has been minimal. Further, most AMs that are synthetically designed in the laboratories are often not pursued extensively with regards to their therapeutic potential. One of the primary reasons for this shortcoming is due to the lack of understanding of the structure-function relationship. For example, multiple allosteric binding sites have been proposed for dfBr that can presumably result in varied functional outcomes at the cellular level. This has however not been explored with respect to other AMs of nAChRs discussed here in the review. Thus, there remains a great potential in understanding how characterizing individual allosteric binding of these drug molecules can provide a resolution on the functional outputs augmenting their therapeutic potential. As this review highlights, nAChRs carry the prospect of being candidate targets to restore inflammatory homeostasis through allosteric regulation (Figure 1). This could potentially fill a gap in the therapeutic targeting of one of the key mediators of inflammatory conditions, thereby addressing a pressing public health issue of the current times.
